# Optimizing Student Success: Leaders’ Perspectives on Advising Practices in Graduate Health Professions Education Programs

**DOI:** 10.5334/pme.1793

**Published:** 2025-08-08

**Authors:** Holly S. Meyer, Anita Samuel, Lauren A. Maggio, Jennifer Cleland, Anthony R. Artino, Emily Scarlett, Paolo C. Martin

**Affiliations:** 1 Department of Health Professions Education, Uniformed Services University of the Health Sciences, US; 2 Department of Medical Education (MED), University of Illinois Chicago, College of Medicine, US; 3 Lee Kong Chian School of Medicine, Nanyang Technological University Singapore, SG; 4 George Washington University School of Medicine and Health Sciences, US; 5 Henry M Jackson Foundation, Department of Health Professions Education, Uniformed Services University of the Health Sciences, US

## Abstract

**Introduction:**

Effective advising is crucial for student success in graduate Health Professions Education (HPE) programs. However, the implementation and impact of advising remain underexplored, particularly from the perspective of program leaders. This study explores how HPE program leaders conceptualize advising practices that support student success.

**Methods:**

We conducted a qualitative narrative inquiry study to explore worldwide leaders’ perspectives on advising in graduate HPE programs. Fifteen program leaders across six World Health Organization regions were purposively sampled and interviewed using a semi-structured approach. Thematic analysis, informed by developmental advising theory, was employed to identify key advising strategies and challenges.

**Results:**

Findings revealed that leaders conceptualize advising in terms of learners’ academic advancement, personal development, and community building. Leaders largely prioritized clear communication, goal setting, and holistic student support within their programs’ advising systems. The advisor-advisee relationship mattered to leaders. They highlighted challenges that might influence this relationship: faculty workload, role conflicts, and the need for structured advising models.

**Discussion:**

This study underscores the critical role of advisors in HPE programs, revealing that effective advising involves both academic guidance and social support. It highlights a gap in the existing literature on how program leaders in graduate HPE programs conceptualize and support advising, and advocates for the expansion of developmental advising that addresses not only administrative tasks but also the broader developmental needs of students, such as career advancement and personal growth. The findings suggest that HPE programs support student success by fostering holistic advising practices and professional development for advisors.

## Introduction

In graduate education, effective advising helps students navigate coursework, research, and career development while supporting their personal and professional growth. Advising is a key component in graduate Health Professions Education (HPE) programs, which include certificate, master’s, and doctoral programs that prepare healthcare professionals (e.g., medical students, dentists, nurse practitioners, and physicians) for roles in teaching, research, and leadership.

Graduate HPE advising involves an assigned individual, generally a faculty member, guiding students through program requirements, including coursework, research, and practical experiences. Many advisors also provide career support, helping students with job applications, job placement, and work-life balance. However, despite its centrality, the implementation of advising in graduate HPE programs remains underexplored [1]. A key distinction is that advising typically focuses on academic and administrative guidance, whereas mentoring encompasses broader personal and professional development.

Effective advising involves key functions such as providing advisees with reliable information, facilitating professional socialization, and supporting career advancement [2]. Thus, educators have turned to the developmental advising model, which integrates academic and personal support, to characterize advising [3,4]. Developmental advising extends beyond administrative guidance, positioning advisors as mentors who facilitate students’ academic and personal growth through a structured yet flexible framework. Rooted in constructivist learning theory, this model aligns with holistic student development and is particularly relevant in HPE contexts, where students navigate complex career transitions and professional identity formation [3,5].

While some research has explored advising structures in HPE programs, a critical gap exists [1] in understanding how program leaders conceptualize advising practices and their influence on student success. Faculty advising in graduate HPE programs occurs within complex institutional and personal constraints. Advisors often juggle competing responsibilities, including research, teaching, and clinical duties, which can limit the time and attention available for student support [7]. Many advisors also lack formal training in advising or mentorship, resulting in variability in the quality and consistency of advising practices [10]. For programs serving part-time or distance learners, maintaining strong advisor-advisee communication and relationships can be particularly challenging [6,8,9]. Although some research has examined digital tools and advising visibility [11,13], there is limited understanding of how program leaders—those responsible for shaping advising environments—perceive and respond to these challenges. This study addresses that gap by exploring how leaders conceptualize advising in ways that promote student success in graduate HPE contexts.

As graduate HPE programs expand [12], understanding how leadership shapes advising effectiveness is essential. In this study, leadership strategies refer to the approaches and actions that program leaders use to shape advising structures, support faculty development, and influence student advising experiences. Without clear leadership strategies, advisors may struggle to balance advising with other professional obligations, potentially limiting the quality of student support. To fill this gap, we examine how HPE program leaders define, implement, and assess advising practices. Specifically, we address the following research question: “How do graduate HPE program leaders conceptualize effective and ineffective advising practices, and how do these perceptions influence their support for student success?”

Although developmental advising is well-established in higher education, its application in graduate Health Professions Education (HPE) remains underexplored. HPE programs often serve diverse, mid-career professionals—including clinicians, educators, and administrators—who bring varied goals, professional identities, and learning trajectories. These programs are frequently offered part-time, online, or across geographic regions, posing unique relational and structural challenges for advising. As such, it cannot be assumed that developmental advising practices, developed largely in traditional undergraduate or residential graduate contexts, will translate seamlessly. This study addresses that gap by examining how advising is conceptualized and adapted by leaders within the distinctive landscape of graduate HPE.

By identifying leaders’ perspectives on advising strategies, we aim to provide actionable insights that improve faculty support and optimize student outcomes in graduate HPE education. While this study focuses on advising practices, it centers specifically on how program leaders understand, support, and influence these practices within their programs. Rather than examining formal leadership strategies in a policy sense, we explore how leaders’ values, decisions, and experiences shape the advising environments they help create. In doing so, we aim to understand the leadership role in advising as it is enacted through everyday practices and perspectives.

## Methods

### Study Design and Participant Recruitment

We employed a narrative inquiry approach, grounded in the understanding that individuals make sense of their lives through stories [13]. This approach was appropriate for exploring how HPE program leaders interpret and give meaning to their advising experiences within complex institutional contexts. Our interview guide was designed to elicit narrative responses—for example, by prompting participants to describe specific advising experiences, challenges, and approaches to supporting student success.

We conducted inductive coding based on these participant narratives, allowing patterns to develop directly from the data. After initial coding and theme development, we drew on Creamer and Creamer’s developmental advising framework to help interpret and organize our findings. Their model supported our understanding of advising as a holistic practice and provided a conceptual structure for synthesizing overlapping concepts into three broader thematic categories: academic advancement, personal development, and community building.

While we used thematic analysis [14] to identify patterns across interviews, the narrative inquiry framework helped guide our attention to the context, sequence, and meaning-making within leaders’ accounts. This approach allowed us to preserve participants’ stories while analyzing recurring themes across them. By combining these methods, we aimed to balance depth and coherence in understanding how leaders conceptualize advising in graduate HPE programs.

We included HPE programs offering master’s and/or PhD degrees. An extensive website search identified 108 eligible programs across the six World Health Organization (WHO) regions: Americas, Europe, South-East Asia, Western Pacific, Africa, and Eastern Mediterranean. To achieve proportional representation, we employed purposive sampling, recruiting participants in batches. Initial recruitment targeted participants from a prior related study [15]. We then focused on underrepresented WHO regions to ensure global representation (Table 1). A team member contacted each program up to three times, but no eligible leaders from the Eastern Mediterranean Region (EMR) responded. We selected program leaders as participants because of their unique position to reflect on advising at both the structural and personal levels. Notably, 12 of the 15 leaders also served directly as faculty advisors within their programs. This dual role allowed them to share insights drawn not only from administrative or leadership responsibilities but also from firsthand advising experience, offering a comprehensive view of advising practices in graduate HPE settings.

**Table 1:** Recruitment of leaders of graduate Health Professions Education programs across the World Health Organization (WHO) regions.


WHO REGIONS	COUNT OF PROGRAMS THAT HAD WEBSITES	INTERVIEWED

African Region	5	1 (17%)

Eastern Mediterranean Region	12	0 (0%)

European Region	33	3 (11%)

Region of the Americas	41	6 (15%)

South-East Asian Region	3	1 (33%)

Western Pacific Region	14	4 (29%)

Total	108	15 (14%)


We determined data sufficiency based on geographic distribution and data richness, as per LaDonna et al. [16]. In total, we interviewed 15 HPE program leaders. Leaders were selected as the initial focus to understand advising within programmatic structures, with plans for subsequent studies involving advisors and students to ensure a comprehensive perspective.

### Data Collection

We conducted one-on-one interviews using a semi-structured interview guide developed from literature [17] and our experience as graduate advisors and graduates of HPE programs. HS piloted the interview guide and, based on that experience, all authors collaborated to refine and finalize the guide. The guide was designed to capture comprehensive coverage of advising practices, including leadership roles, program structure, advising models, barriers, and ideal advising frameworks.

Interviews, conducted in English by HM and PM in Spring 2023 via video conferencing, lasted 45–75 minutes and were video-recorded. Recordings were transcribed verbatim and de-identified. The Institutional Review Board of the Uniformed Services University approved the study (Case#: DBS.2022.348).

### Reflexivity

Our research team comprises current and former HPE program advisors and leaders across North America, Europe, and Asia. This insider perspective allowed for a nuanced understanding of advising structures, while our systematic analytical approach ensured objectivity. We adopted an emic-etic perspective [18], leveraging insider knowledge while maintaining critical distance through developmental advising theory.

Each author has extensive experience in advising and mentoring within graduate HPE programs:

- HSM: Vice Chair of Student Affairs, advisor for 10 years, oversees faculty advisors for 250+ learners annually.
- AS: Assistant Dean for Graduate Education, Vice Chair of Distance Learning, advising over 100 graduate students.
- LAM: Professor and Director of Research, advised and mentored 50+ PhD and MHPE students.
- JC: Led a PhD program (2020–2024), supported a team advising 100+ learners, and mentored 27 PhD/MSc students.
- ARA: Associate Dean for Educational Research and Co-Director of the Academy of Education Scholars, advising 41+ graduate students and faculty.
- ES: Provided research assistance, including methodology support and conceptual overview of the project, while drawing on the experience of being advised in a graduate program.
- PM: Assistant professor in the department of HPE, where he has advised 15 PhD/MHPE learners and 45 HPE certificate learners. Also, a fellow of programs designed for minoritized pre- and early-career faculty. Serves on the steering committee for the MedEdSCHOLAR mentorship program for early career faculty in academic medicine.

### Data Analysis

Narrative inquiry was employed to capture diverse perspectives on advising within graduate HPE programs. We conducted an inductive thematic analysis following Braun and Clarke’s [14] six-phase process: (1) familiarization with data, (2) generating codes, (3) searching for themes, (4) reviewing themes, (5) defining and naming themes, and (6) producing the report.

Initially, HM coded four transcripts, with HM and PM reviewing the preliminary codes. ES coded the remaining 11 transcripts, after which HM and PM refined coding structures. The research team (HM, LM, AS, ES, AA, JC, PM) reviewed coded data and sample transcripts, engaging in iterative discussions to refine themes. Through consensus-building, we identified key links between developmental advising theory and emerging patterns [19].

Developmental advising theory [4] informed our analysis, emphasizing advising as a holistic practice integrating academic, personal, and professional growth. We applied Creamer and Creamer’s [3] model, which extends developmental advising beyond administrative tasks to mentorship-focused support. This framework aligned with our constructivist approach, emphasizing student-centered, co-constructed learning.

During analysis, we initially organized our findings using Creamer and Creamer’s six dimensions of developmental advising: setting career and life goals, building self-insight and self-esteem, broadening interests, establishing meaningful interpersonal relationships, clarifying personal values and lifestyles, and enhancing critical thinking and reasoning. While these dimensions were evident in our data, participants’ descriptions often blended multiple concepts, such as building self-esteem and clarifying values, making it difficult to clearly separate them in practice.

To improve clarity and reduce redundancy, we inductively grouped overlapping dimensions into three broader, integrative categories: academic advancement, personal development, and community building. This reorganization preserved the theoretical foundation while better reflecting the holistic and interconnected nature of the advising practices described. These decisions were made through iterative team discussion to ensure analytic coherence and trustworthiness.

Notably, most participants were both program leaders and active advisors. This overlap allowed the author team to reflect on advising practices through multiple lenses, describing institutional expectations and supports and their strategies and experiences as individual advisors. This dual perspective enriched our understanding of how advising is conceptualized and practiced across graduate HPE programs. This collective expertise influenced data interpretation, ensuring rigorous analysis while mitigating bias through continuous reflexive discussions.

## Results

Fifteen HPE program directors from eight countries and 15 institutions participated in the study. Twelve of the 15 program directors also served as advisors to students within their programs. Because many of the programs’ directors also served as advisors, the distinction between advising practices they support and train their advisors on versus those they personally use became blurred. Both are reported. Throughout the interviews, participants discussed both beneficial and ineffective advising practices and the structure of advising in their program. We have organized their descriptions into three main themes: academic achievement, personal advancement, and career building.

We reorganized Creamer and Creamer’s [3] concepts because the practices leaders described blurred the lines of the six concepts based on how the program used them in their individualized framework. We wanted to emphasize the interconnectedness of these areas in fostering holistic student development and preparing future healthcare professionals. This streamlined framework allowed us to more effectively categorize and analyze the data, highlighting the key areas where developmental advising impacts student growth and success based on leaders’ perspectives. To provide context for our findings, we share participant quotes labeled with a letter (e.g., D would indicate the leader expressed the corresponding statement). Table 2 summarizes the advising practices from the perspective of HPE leaders.

**Table 2:** Advising practices described by graduate health professions education (HPE) leaders.


	ADVISING PRACTICES

Academic Achievement	Know and share academic requirements, curriculum changes, and expectations. Communicate effectively through various channels (group sessions, individual check-ins, and emails). Manage coursework by mapping out programs of study, selecting courses, and assisting with registration. Ensure students stay on track with deadlines and progress tracking. Actively engage students in learning and connecting concepts. Prepare educators with educational theories and skills. Guide research projects with feedback and resources. Refer students to support services and offer additional assistance. Show genuine interest in students’ success. Encourage professional development and facilitate career opportunities.

Personal Achievement	Serve as a critical friend. Engage in supportive, non-hierarchical advising. Collaborate with students, guiding them while allowing ownership of their work. Facilitate joint knowledge-building. Draw from personal experience as a student. Serve as a sounding board and provide advice. Offer insights and feedback based on qualifications. Help students understand strengths and areas for improvement. Encourage belief in their capabilities. Promote new ways of thinking. Support conflict resolution and personal challenges. Enable students’ journeys and goals. Provide career guidance and set realistic academic goals. Offer individualized support. Address non-program-related issues. Show genuine interest in students’ well-being. Assist with accommodations and campus familiarization.

Community Building	Cultivate diversity, inclusivity, and equity. Promote social justice. Value all health professionals’ voices. Encourage active participation and peer engagement. Keep students engaged and motivated. Highlight the importance of a community of practice. Create a warm, empathetic, safe, and inclusive environment. Foster critical thinking and strengths-based narratives. Build trust and rapport with students. Model expected behaviors like effective communication, humility, transparency, integrity, fallibility, vulnerability, and accessibility.


### Academic Advancement

HPE leaders described using various advising practices to support students’ academic advancement. These included understanding and communicating academic requirements, curriculum changes, and program expectations. Participants also described helping students manage their coursework by mapping out their program of study, selecting appropriate courses, and assisting with registration. For example, one participant noted: “The purpose is quite explicit: to guide students to navigate the program, which is highly individualized and self-directed” (K).

Leaders reported that advisors supported academic advancement through communication, deadlines, and portfolio checks. Advisors communicated clearly and effectively through group advising sessions, individual check-ins, and emails. Advisors ensured students stayed on track and made progress toward their goals, sometimes by using deadlines to create a sense of urgency and structure as needed. Programs that used portfolios noted that their advisors helped to document the student’s process via portfolios. Advisors also used practices that directly helped students in their studies by assisting them to connect concepts across courses or contexts and providing knowledge of educational concepts, theories, and skills. Participant H expands on this by stating, “Our goal is often to surface their educational needs, articulate them with them, and then send them in that direction. Maybe sometimes it is directly teaching them about whatever that might be, but often it’s actually directing them in the…providing resources for them to identify the answers, fulfill the knowledge gap or skills gap themselves.”

Some leaders noted that advisors play a role in the student’s research project, helping to guide the student through it and ensure academic rigor. Such assistance shapes research advisory teams, guides the selection of research topics, provides project feedback, and equips advisees with resources. “To take the student through the thesis process from the very beginning, focusing on the research question and putting together a pre-proposal and then a proposal, taking them through their proposal hearing, helping them with IRB, advising during the data collection and analysis, reviewing and critiquing the final thesis write up, taking them through the thesis defense, all of that” (M).

Advisors use multiple strategies to support students broadly in their education. These strategies include helping them to transition to higher-level studies (e.g., a master’s student decided to enroll in a PhD program), encouraging them to make connections or seize opportunities, and sponsoring their educational development. Participant G emphasizes this transition by explaining, “I’ve had some MA students who are now doing a PhD with me in HPE.” Another leader described connecting advisees with the larger community through networking, “we (the program) have got connections.…so we can enable our students to achieve whatever they want to achieve, by connecting them with other people” (F). When advisors cannot directly support students, advisors sometimes refer students to other supports such as mental wellness programs, financial support, or writing centers. Of note, a few programs did not offer any kind of formalized career discussions: “Because not all our participants [students] want to do that…They do not care about a career because it’s not a career they’re building in med ed. They just happen to be teaching because their workplace said they had to” (J).

### Personal Advancement

In HPE, advising practices supported students in their personal advancement. Advisors emphasized the importance of tailored, empathetic advising practices and precise, effective communication to foster active engagement and a strong advisor-advisee relationship. Support extended beyond academics by offering “non-academic support, more mentally supporting students, emotionally supporting them, and helping them manage their workload, study load, work-life balance, family, work, study, life balance” (E). Participant H said, “It’s important that we know our students and that there’s a strong emotional component.” They support the relationship by engaging in practices including facilitating knowledge building together, serving as a critical friend, being more supportive and less hierarchical, and being collaborative while giving students ownership of their work. They could partner beside the student by acting as a sounding board, offering insight based on their qualifications or experiences in the program and providing feedback when needed. Advisors can “learn from students; there is a lot of co-learning. It also maintains my relationships with clinicians, which are important for my program of research. So, a lot of the MA students that I supervise in HPE are clinicians whom I do research with” (G).

Many advising practices revolve around student goals. HPE advisors help promote goal setting, enable the student’s journey, provide opportunities for career discussions, manage professional expectations, assist with a student’s personal goals, and adapt to support students based on their unique needs, goals, and interests. Sometimes, enabling the student’s journey took on an advocacy and championing tone, “university policies and procedures…most of the time they’re an irritant…You have a good student; they’ll go through and they’ll put lots of barriers in their way, as often happens. So, a lot of the time I spend advocating for students, whether it’s for admissions or they’re not moving forward well, but there’s a reason, give them a break type of thing” (C).

Other practices revolved around developing the students and their insight by promoting their understanding of their strengths and areas for improvement, turning weaknesses into learning opportunities, encouraging students to believe in their capabilities, promoting new ways of thinking, helping them with conflict resolution, and helping them work through personal challenges.

Advisors also had to engage in other practices to assist the students’ advancement. These included dealing with non-program-related issues, providing guidance on accommodations and campus familiarization, showing genuine interest in the student’s well-being, and being aware of the challenges of distance learning. Much like developmental and academic advisors, HPE advisors helped holistically support the students’ growth with practices that encouraged a close, less hierarchical relationship in which they could also learn alongside the student.

However, some programs supported advising without any closeness, “I never saw it as a mentor or coaching role, because I don’t think I had the level of intimacy in the relationship” (J). Sometimes, the context of the program prevents closeness because advisees come from established professional careers. One leader stated, “[I] had a student who had some really, really significant mitigating circumstances, and we tried to get him to go to see the pastoral tutor [advisor]. He wouldn’t see him because he said, this person knows me in my professional context, and I don’t want him to know that I have this going on in my life. And so, it was, it actually mattered quite a lot who this person was at that point; it wasn’t sort of a neutral thing” (O).

### Community Building

HPE advisors play a vital role in fostering inclusive academic communities by encouraging diversity, equity, and active participation. Advisors create spaces for students to express ideas, challenge dominant narratives, and engage collaboratively. Participant N emphasized, “We strive to consider diversity and inclusion in all aspects, from research topics to advising approaches.” Advisors build trust and rapport, fostering critical thinking and encouraging strengths-based development. Participant I noted, “Many students feel self-critical, and our role is to build their confidence and help them believe in their capabilities.” Advisors also help students navigate professional challenges while ensuring they feel supported within the academic community. Participant L shared, “Advisors balance creating opportunities for students to participate in the discipline while giving them space to explore their ideas.” Additionally, A highlighted that “modeling expected behaviors and maintaining accessibility is key to building student trust and engagement.”

Moreover, advisors emphasize creating a warm, empathetic environment where students feel valued and understood. F mentioned, “Advisors foster a sense of belonging by consistently checking in with students and addressing their individual needs.” H added, “Supporting students emotionally and academically creates a tight-knit community that thrives on mutual respect.” As G highlighted, encouraging students to pursue personally meaningful research topics “helps them stay motivated and connected to the broader academic community.”

## Discussion

Our study identified a range of leaders’ perceptions of advisor-advisee practices within graduate HPE programs. These findings illustrate how leadership strategies, such as setting advising expectations, advocating for resources, or shaping advisor roles contribute to advising effectiveness. Data suggest effective advising encompasses academic and personal support, such as assisting with course selection and providing substantial emotional support. The advisor’s role in creating a supportive community and learning environment was also crucial in helping students develop a sense of belonging and mutual respect. The key findings focused on advisors’ need to have up-to-date programmatic knowledge, provide individualized support, and prepare students for their professional careers [20,21].

### Expanding Developmental Advising in Graduate HPE Programs

Building on existing literature, we highlight the need for developmental advising in graduate HPE programs. While previous research has emphasized administrative aspects of advising, such as course selection, this study underscores the importance of addressing the broader developmental needs of students, including career advancement and professional development [5,22]. The findings align with developmental advising theory, which views advising as a holistic process that fosters students’ intellectual, personal, and career growth [3,4]. Our study extends this theory by illustrating how program leaders perceive the integration of developmental advising principles into HPE, where students often balance academic progression with professional responsibilities.

These findings align with the broader literature on student support in higher education. King [23] suggests effective advising should be structured yet adaptive, incorporating mentorship-style support, while fostering student autonomy [23]. Embracing the distance learning format of graduation HPE programs, Samuel et al. [11] highlight the effectiveness of online academic advising strategies tailored to high-achieving professionals. Their findings demonstrate how advising can extend beyond in-person interactions to ensure continuous professional support. The online representation of HPE programs was studied by Schermerhorn et al. [15] noting their advising practices online. Advising visibility and accessibility are crucial factors in student engagement, expectation setting, and program satisfaction. These insights reinforce the argument that HPE programs must evolve advising models to accommodate changing student needs, mainly through technological enhancements and structured approaches.

### Enhancing Advisor Development and Holistic Support in Graduate HPE Programs

Graduate HPE programs should prioritize ongoing training for advisors to ensure they remain current with program requirements and policies [10,21]. Professional development is essential for delivering individualized support and preparing students for their careers. One potential solution is adopting apprenticeship advising models emphasizing mentorship and hands-on learning experiences [24]. Given the diverse professional backgrounds of HPE students, training for advisors is necessary.

Additionally, institutions should revise their policies to support holistic advising practices beyond administrative functions. Developmental advising frameworks, such as Creamer and Creamer’s [3] model, emphasize fostering an inclusive and engaging advising environment that supports academic and personal growth. Institutional investment in training programs and dedicated advising resources could help ensure advisors have the skills and knowledge to effectively fulfill these roles [2]. Moreover, future research should explore how program leaders can establish clear expectations for advising, develop effective training methodologies, and implement systems to recognize and promote advisors [20,25]. Understanding the role of institutional support, particularly in tenure and promotion, could provide valuable insights into incentivizing effective advising practices [8,26,27].

### The Role of Digital Tools in Advancing Advising Practices

As digital advising tools and learning analytics become increasingly prevalent, program leaders should consider integrating these resources to enhance advising effectiveness. Tekian, Dekhtyar, and Park [12] emphasize the importance of international trends in health professions education, suggesting that advising must incorporate best practices from a global perspective. Digital advising platforms offer real-time feedback on student progress and provide personalized recommendations based on predictive analytics. HPE programs can create more flexible, student-centered advising models by integrating technology into advising structures.

For example, Schermerhorn et al. [15] suggest that graduate HPE programs improve how they communicate advising expectations and resources to students, emphasizing the need for transparency and ensuring students can easily access information. Leaders could explore how various advising tools (artificial intelligence-driven advising platforms, interactive advising dashboards, and peer mentoring networks) can complement existing advising structures to provide a comprehensive support system for students.

### Policy Implications

HPE programs should reconceptualize advising as an integrated function that blends academic, personal, and professional support. Rather than treating advising as an auxiliary component of graduate education, institutions should view it as a core function influencing student success, retention, and career readiness. Program leaders may explore policies that formalize advisor training and implement initiatives that could ensure that advisors receive professional recognition for their work in student development.

### Strengths, Limitations, and Future Directions

Strengths of the study include the diverse perspectives collected from HPE program leaders across multiple global regions, which provide rich insights into advising practices. Applying developmental advising theory as a guiding framework ensures that the analysis is rooted in a well-established student support and development model. Additionally, qualitative narrative inquiry allowed in-depth exploration of leaders’ experiences and perspectives.

While diverse, the sample size of 15 leaders is relatively small and may not capture all possible perspectives. Researchers could enhance the understanding of global advising practices in HPE by expanding research to include non-English-speaking regions and various program types, since the recruitment protocol builds on the work of a previous study. Furthermore, the study focused primarily on master’s and PhD programs, which may not fully represent advising practices in other graduate HPE programs.

Future research should examine how developmental advising influences long-term student success, including career trajectories, job placement, and professional growth. Additionally, exploring the perspectives of students and advisors themselves would complement this study’s leadership-focused findings, offering a more comprehensive understanding of advising dynamics in graduate HPE programs.

## Conclusion

Findings emphasize advisors’ critical role in providing academic and personal support within graduate Health Professions Education (HPE) programs. The results further highlight the need for up-to-date programmatic knowledge and individualized guidance. Expanding developmental advising to address students’ broader career and personal development needs, a practice that remains underexplored in graduate HPE programs, is needed. Thus, program leaders should prioritize regular professional development opportunities for advisors focused on current trends in the field, advising best practices, and student mental health; implement a holistic advising model that includes regular check-ins, career counseling, and support for personal well-being; and allocate funding for student memberships in professional organizations, attendance at conferences, and networking events with alumni, better preparing them for their careers.

## Disclaimer

This work was prepared by a civilian employee of the US Government as part of the individual’s official duties and therefore is in the public domain. The opinions and assertions expressed herein are those of the author(s) and do not necessarily reflect the official policy or position of the Uniformed Services University of the Health Sciences, the United States Department of Defense, or the Henry M Jackson Foundation for the Advancement of Military Medicine, Inc.
